# Local Activity and Selectivity Hotspots in Cu‐Pt Model Thin‐Film Electrocatalysts for Oxygen Reduction

**DOI:** 10.1002/smtd.202502169

**Published:** 2026-01-28

**Authors:** Lewin V. Deville, Rico Zehl, Luca Saluta, Qingdian Liao, Peter M. Schneider, Tobias Piotrowiak, Benedikt Kohnen, Ellen Suhr, Alfred Ludwig, Aliaksandr S. Bandarenka

**Affiliations:** ^1^ Physics of Energy Conversion and Storage, Physics Department Technical University of Munich Garching Germany; ^2^ Institute For Materials Chair For Materials Discovery and Interfaces Ruhr University Bochum Bochum Germany; ^3^ Catalysis Research Center TUM Garching Germany

**Keywords:** CuPt, EC‐STM, electrocatalysis, oxygen reduction reaction, platinum

## Abstract

While state‐of‐the‐art alloy catalysts for the oxygen reduction reaction (ORR), a key process for future sustainable energy provision, rely on platinum‐rich materials, alloys containing less noble metals may play an increasingly important role. In particular, Cu–Pt systems are among state‐of‐the‐art electrocatalysts for O_2_ electro‐reduction, demonstrating high activity and selectivity for the four‐electron pathway. This study explores the behavior of Cu–Pt model thin film alloy catalysts using electrochemical scanning tunneling microscopy (EC‐STM), a technique capable of detecting active sites and areas for surface catalytic processes under reaction conditions. Our findings indicate that the nature of active centers changes depending on whether the final product is H_2_O or H_2_O_2,_ which can also be generated in parallel. Active centers are located on the (111) terraces for the four‐electron ORR and shift to step defects if the hydrogen peroxide generation starts. On the other hand, the grain boundaries do not seem to contribute to the sample activity. These findings can be used in designing the shape of nanoparticles for improved nanostructured materials for energy applications.

## Graphical abstract

A Cu‐Pt model thin film catalyst is investigated via electrochemical scanning tunneling microscopy. This technique enables in‐operando active site detection on a nanometer scale. It is shown that the precise location of the active sites changes depending on the selectivity of the oxygen reduction reaction.

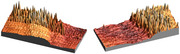

## 1 Introduction

As global environmental concerns increasingly intensify, countries use renewable energy provision schemes [1, 2, 3, 4]. The so‐called “green” hydrogen has emerged as an attractive energy carrier due to its high energy density [5, 6]. Hydrogen also plays a pivotal role as an industrial feedstock for synthesizing chemicals such as ammonia and methanol [7], and its utilization in fuel cells and metal‐air batteries has revolutionized transportation and stationary power generation [8, 9, 10]. However, despite these advances, the overall efficiency of energy conversion, e.g., from water electrolysis to fuel cell operation, is still limited by the sluggish kinetics of the oxygen reduction reaction (ORR) [11, 12, 13]. Catalysts are, therefore, crucial in reducing these energy losses, making their optimization a central focus of current research [14].

The catalytic performance is intimately linked to binding properties and the electronic structure of the catalyst surface [15, 16]. Platinum (Pt) is widely regarded as the benchmark catalyst for the ORR owing to its near‐optimal binding of reaction intermediates [17, 18]. For the ORR, Pt demonstrates one of the highest activities in acidic conditions, as it also enables efficient O_2_ dissociation and subsequent reduction via the preferred four‐electron pathway, minimizing the formation of H_2_O_2_ by‐products [19, 20]. However, Pt‐based catalysts also face several practical challenges. For instance, Pt performance is significantly influenced by the pH of the electrolyte, which can lead to different reaction pathways and alter the adsorption characteristics of oxygenated intermediates [21]. In alkaline media, pH‐dependent effects are reproducibly observed for the ORR in the presence of various alkaline metal cations [22, 23, 24]. Moreover, Pt suffers from significant overpotential losses (typically exceeding 200 mV) in the ORR, which reduces the overall energy conversion efficiency [25]. Additionally, reducing the amount of scarce Pt remains a significant challenge for large‐scale applications [26].

Current research focuses on modifying Pt‐based catalysts to address these challenges and enhance activity and durability. Alloying Pt with other metals, incorporating non‐metal materials, and constructing heterojunction interfaces can modify catalytic centers through the so‐called electronic and strain effects, fine‐tuning the adsorption energies of key intermediates [12, 18, 27]. For instance, Fe–Pt alloy catalysts demonstrate enhanced ORR activity and durability by modifying the electronic structure. The incorporation of Fe leads to an electron transfer to Pt and a subsequent downshift in the d‐band center, optimizing the oxygen binding energy for efficient O_2_ reduction while minimizing peroxide formation [28, 29]. Moreover, Fe also improves durability by mitigating Pt dissolution and suppressing surface oxidation [30]. In contrast, Pt–Ru alloy catalysts have been shown to significantly boost HER performance in alkaline conditions by facilitating the water dissociation step; Ru sites promote OH‐adsorption and lower the energy barrier for water splitting, thus accelerating hydrogen evolution in alkaline solution [31]. NiFe nanoparticles can also provide optimal OH‐adsorption sites, facilitating water dissociation [21, 32]. While PtNi catalysts may differ in their specific Ni surface content, this class of materials ranks among the closest to the optimal point on the volcano plot, leveraging strain effects to enhance catalytic performance [33, 34, 35]. Pt alloys containing Co can also exhibit high ORR efficiencies, particularly when the Co content is in the range of 25–40% [36, 37]. Additionally, Gd–Pt and Pt‐Y alloys offer promising catalytic properties; however, the limited availability of these rare‐earth elements could present challenges for large‐scale industrial implementation [38, 39, 40]. More broadly, alloying Pt with complementary metals can enhance atomic utilization and reduce material costs without compromising catalytic efficiency [41, 42, 43, 44].

Cu–Pt alloy systems are among the extensively studied catalytic systems for HER and ORR [45, 46, 47, 48]. Alloying Pt with Cu modifies the electronic structure of Pt through synergistic effects, tuning the d‐band center to optimize oxygen binding for the ORR while also decreasing Pt loading and potentially enhancing catalyst stability [49, 50, 51]. In these Cu–Pt systems, Pt typically provides active sites for hydrogen adsorption and oxygen reduction, while Cu alters the electronic properties of the surface, thereby tuning the adsorption energies of key intermediates. Density functional theory (DFT) calculations show that Cu incorporation shifts the Pt d‐band center toward the Fermi level, enhancing hydrogen adsorption for the HER while optimizing oxygen binding for the ORR [52]. However, the specific nature of these active sites, whether they are associated with low‐coordination defect sites or specific crystalline facets, remains an area of ongoing research. Despite the theoretical advantages suggested by computational studies, direct experimental evidence elucidating the nature and distribution of active sites in Cu–Pt alloys remains scarce [53]. This study aims to further understand such Cu–Pt synergistic systems by studying their active sites during different reaction conditions to widen the scientific horizon within this specific field of electrocatalysis.

Recent advances in operando tunneling noise detection via an electrochemical scanning tunneling microscope (n‐EC‐STM) offer a unique opportunity to characterize active sites [54, 55]. n‐EC‐STM is an approach specialized in detecting fluctuations in the tunneling current caused by electrochemical reactions at active centers and regions. Switching the electrochemical reaction “on” and “off” enables the direct visualization and correlation of noise signals with the locations of electrochemical activity. Previous studies have successfully applied this technique to systems such as Pt‐based lanthanide alloys [56] and Pt_3_Ni catalysts [42], as well as various other catalytic systems [29, 57, 58, 59, 60]. Moreover, the data processing approach proposed recently allows for a quantitative comparison of site activity, enabling a more precise identification of the primary active sites [61]. Within the scope of this study, n‐EC‐STM is employed to investigate the active sites of model Cu–Pt alloys during the ORR processes, thereby revealing variations in active site characteristics across different reactions, providing direct experimental evidence to validate theoretical models, and offering insights for the rational design of high‐performance, cost‐effective electrocatalysts.

## 2 Experimental Section

The analyzed Cu–Pt sample was deposited by magnetron co‐sputtering in a commercial four‐cathode sputtering system (ATC 2200, AJA International). For thin film deposition, two cathodes opposing each other were used. Pt and Cu were sputtered from elemental 4‐inch diameter targets with 99.99% purity (Pt: MaTecK GmbH, Cu: Sindlhauser Materials GmbH). Sputtering was carried out in a non‐reactive Ar atmosphere, with a constant process pressure of 0.4 Pa and a gas flow of 80 sccm Ar. Cu was sputtered with a power density of 1.36 W/cm^2^ and Pt with a power density of 1.23 W/cm^2^. As substrate, an oxidized 4‐inch (100)‐Si‐wafer with 500 nm of thermal SiO_2_ was used. The 720 s long deposition was carried out at room temperature without intentional heating of the substrate.

For subsequent heat treatment, a modified tube furnace from Thermconcept (model ROK 150/500/12) was used. The sample was heat‐treated at 900°C for 24 h in vacuum (10^−6^ mbar). After heat treatment, the furnace was turned off, and the sample was continuously cooled down under vacuum to room temperature.

The composition of the investigated film was measured using energy dispersive X‐ray spectroscopy (EDX) in a scanning electron microscope (SEM, JEOL 5800 equipped with an Oxford INCA X‐act detector). The acceleration voltage was set to 20 kV, with a magnification that provided a field of view (FOV) of approximately 400 µm × 600 µm. The composition of the measured area is an averaged value and is considered homogeneous. However, this value does not reflect the surface composition of the thin film. Measurements of the respective surface composition were done using X‐ray photoelectron spectroscopy (XPS).

To obtain crystallographic information, X‐ray diffraction (XRD) was performed with a Bruker D8 Discover using a Vantec‐500 2D detector in Bragg–Brentano geometry with an Incoatec High Brilliance Iµs Cu Kα X‐ray source (*λ* = 0.15418 nm). Thereby, three 2D frames were collected at the θ/2θ steps: 12.5°/25°, 22.5°/45°, and 32.5°/65 with a step time of 40 s for each frame. For phase identification, a 1D diffractogram is derived by integration of the merged 2D frames.

The X‐ray analysis was complemented by the CALPHAD methodology using the Thermo‐Calc Software version 2025b [62] and the Thermo‐Calc Software TCNOBL3 Noble Metal Alloys Database [63]. By calculation of the equilibrium condition of the sample's chemical composition, measured via EDX, phases for matching with the XRD results were pre‐selected.

For the characterization of the sample's microstructure, a SEM (Jeol JSM‐7200F) with 20 kV acceleration voltage and 10.1 mm working distance (WD) with a lower electron (secondary electron) detector (LED) was used.

All STM images were collected using a MultiMode SPM device (Veeco Instruments) in combination with a Nanoscope III feedback controller (Veeco Instruments) and a Universal Bipotentiostat (Veeco Instruments). All EC‐measurements were conducted in a 0.1 m HClO_4_ (Merck Suprapure, 70%) electrolyte that was mixed with ultrapure water (0.0055µS/cm, Stakpure), together with an SSC Reference Electrode (Xylem Analytics) and a Pt Counter Electrode (99.99%, MaTeck). The STM tips were produced mechanically using a PtIr alloy (80% Pt, 20% Ir, 99.9+%, MaTeck) and Apiezon Wax (Plano GmbH) that was used to partially isolate the tip from the acidic electrolyte.

The data analysis was performed using the WSxM 5.0 Develop Software [64] and a self‐written analysis tool [61]. All electrochemical cleaning processes used a VSP‐300 Potentiostat (BioLogic), and the annealing processes took place in a tubular oven (Heraeus Instrument Group). The XPS spectra of the Pt_3_Cu sample were collected on a SPECS setup (SPECS XR50 X‐ray source, SPECS PHOIBOS 150 hemispherical analyzer, and SPECS spectrometer; Specs, Germany) using a monochromatized Al‐Kα source (1486.7 eV). XPS spectra were recorded after electrochemical characterization through EC‐STM and after Ar sputtering. Ar sputtering was performed under pressure below 5 x 10^−6^ mbar with a 1 kV acceleration voltage to achieve a constant ion flux of 6.8 µA. All spectra were acquired in an ultra‐high vacuum chamber at an operating pressure below 1 x 10^−9^ mbar. The data was analyzed with the Casa XPS software (Version 2.3.24PR1.0). The binding energy scale correction was done using the C═C peak of the C 1s spectrum at 284.0 eV.

## 3 Results and Discussion

**FIGURE 1:**
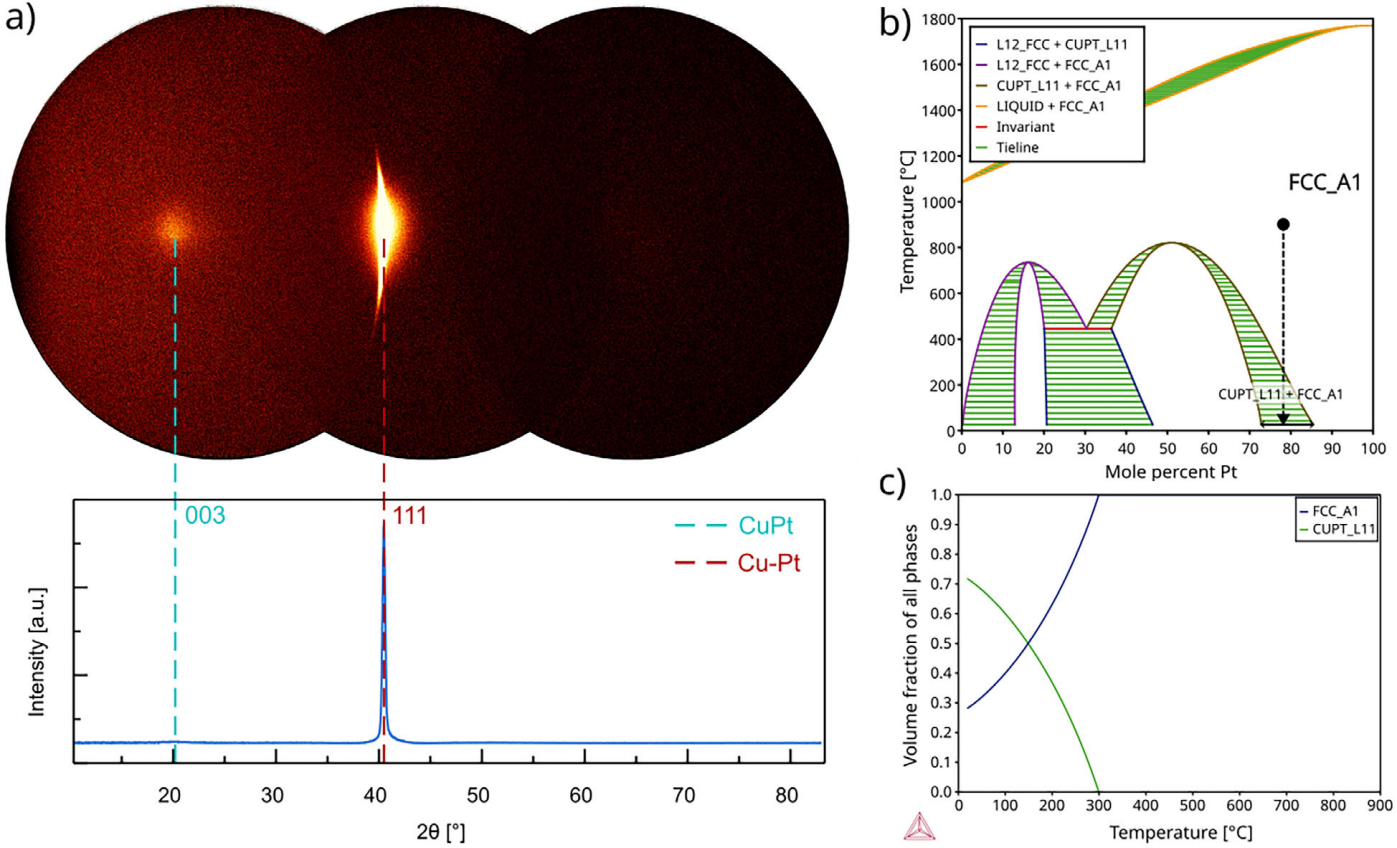
Phase analysis by X‐ray diffraction and Thermo‐Calc calculations. a) 2D diffraction data in the top and derived 1D diffractogram in the bottom. Identified peaks are indexed and highlighted with a dashed line in the respective color corresponding to the phase, i.e., CuPt intermetallic and Cu–Pt solid solution. b) Calculated Cu–Pt binary phase diagram by Thermo‐Calc. The idealistic cooling in equilibrium from 900°C for the sample's EDX composition is shown via a black dashed line and a black circle. c) Volume fractions of phases vs. temperature in °C was calculated for thermodynamic equilibrium for the sample's EDX composition. At around 300°C, the intermetallic CuPt phase appears and overtakes the volume fraction of Cu–Pt solid solution at around 150°C. However, experimental results suggest that equilibrium was not reached, since the Cu–Pt solid solution is the pre‐dominant phase in the sample.

**FIGURE 2:**
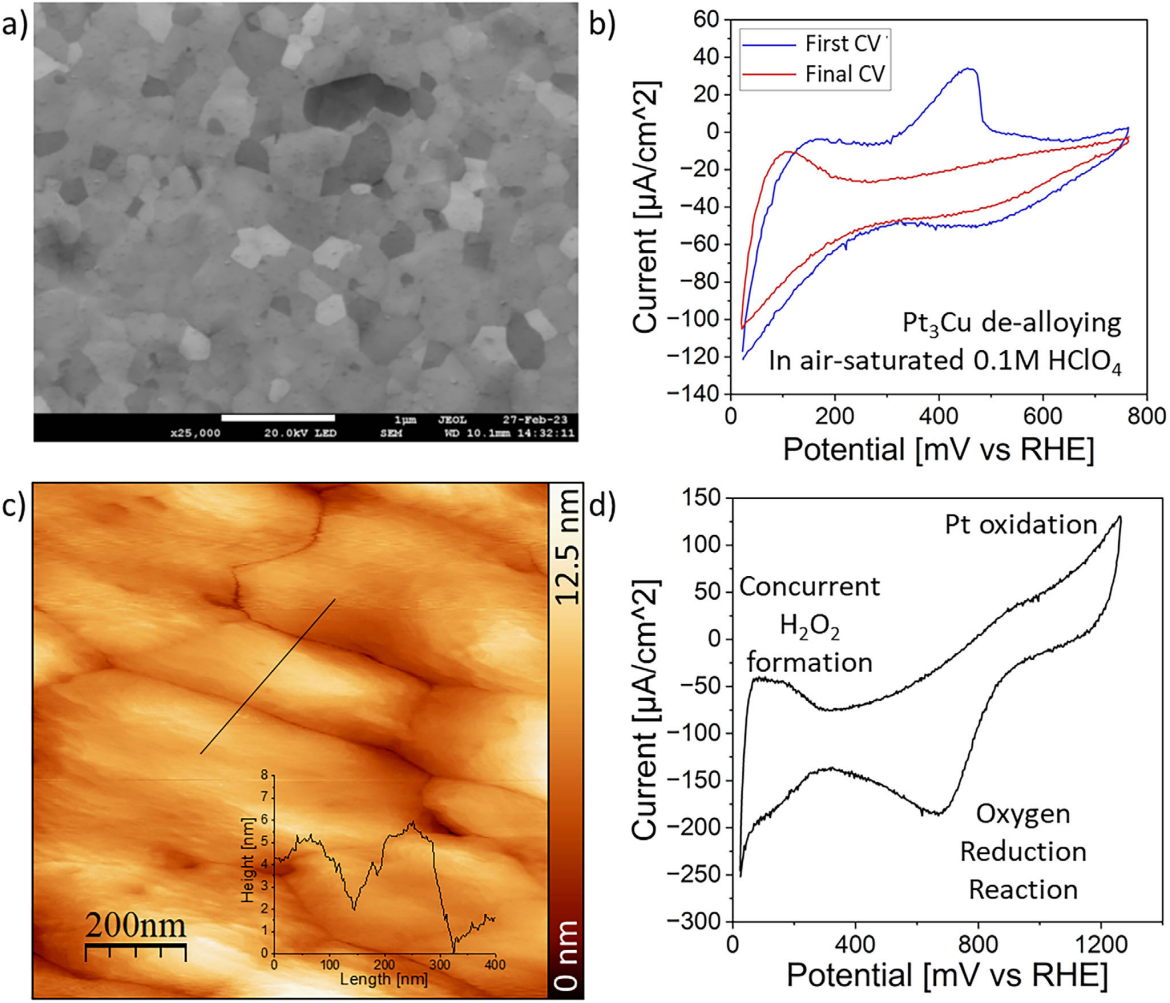
Characterisation of the Cu‐Pt surface before and after the electrochemical dealloying process. a) A typical SEM image of the as‐synthesized sample. b) Cyclic voltammograms showing the first cycle (blue) and the stable cycle (red) recorded in air‐saturated 0.1 m HClO_4_. c) Sample overview after electrochemical dealloying and annealing. d) A typical cyclic voltammogram of the annealed and de‐alloyed sample in air‐saturated 0.1 m HClO_4_.

Prior to any electrochemical STM measurements, the as‐synthesized surface composition, after thin film deposition and annealing at 900°C for 24 h, both from a morphological and chemical perspective, had to be validated. Moreover, the crystallographic characterization via XRD was also done before electrochemical STM measurements. Figure 2 reports the overview of the characterization and electrochemical pretreatment of the annealed Cu–Pt sample. The alloy's microstructure was examined by SEM, a typical image is shown in Figure 2a. A microstructure composed of grains with sizes of 200–400 nm, predominantly with (111)‐orientation, was observed. The chemical composition of the Cu–Pt alloy in that area was measured first via EDX, giving 22.9 at.% Cu and 77.1 at.% Pt as a result. XRD analyses, depicted in Figure 1a, showed that the sample largely consists of a Cu–Pt solid solution with a cubic crystal system and face‐centered cubic (fcc) lattice. The 111 peak at 40.56° 2θ matches with #1965497 (cF4, Fm‐3m) of Pearson's Crystal Data (PCD). However, the results revealed hints of intermetallic CuPt precipitates in the analyzed region, by a very broad (003) peak of low intensity at around 20° 2θ, which was corroborated by Thermo‐Calc calculations. The (003) peak was matched with reference #1819819 (hR6, R‐3m) of PCD. Although Thermo‐Calc calculations predict a higher phase fraction of intermetallic phase (CUPT_L11) compared to the solid solution (FCC_A1) in the equilibrium state, see Figure 1b,c, we assume that the undefined cooling rate after annealing was enough to hinder diffusion in the small thin film volume sufficiently, such that the solid solution is still the predominant phase in the sample. Since electrochemical STM measurements are very localized, the influences of the intermetallic phase on the electrochemical results can be excluded.

To prepare the sample for electrochemical STM investigations, further pretreatment, such as electrochemical dealloying, had to be conducted in order to get the sample into an electrochemically stable state. The Cu‐Pt film was brought into contact with an air‐saturated 0.1 M HClO_4_ electrolyte and subjected to cyclic voltammetry in the range of 0.06–0.8 V/RHE at 50 mV/s. Figure 2b reports the data during the first and last cycles of this electrochemical procedure. During the initial cycles, the current response is characterized by an irreversible peak in the range of 0.3–0.5 V/RHE, which diminishes quickly over time until it is no longer detectable. This phenomenon is typical for Pt alloys containing non‐noble metals, such as copper [65]. Such an electrochemical dealloying process induces selective stripping of the non‐noble metal (Cu) from the surface. Hydrogen underpotential deposition (HUPD) manifests itself in the range of 0.06–0.3 V/RHE in the final cycles. These characteristic Pt voltammetric features [66] are barely visible before the electrochemical dissolution of Cu from the surface (Figure 2b). This behavior suggests the formation of a Pt‐rich surface after the dealloying process. The sample was then annealed at 600 K for 2 h in an Ar‐H_2_ atmosphere. The choice of these annealing conditions aims to provide enough energy for surface Pt atoms to diffuse and reduce the superficial stress while avoiding significant phase changes during the process. Figure 2c illustrates the STM scan of the topography of the annealed surface. The microstructure was restored after annealing, showing domains that range from 300 nm to 700 nm in size while keeping an otherwise smooth surface. Furthermore, the electrochemical stability of the sample was tested in the potential window of 37 to 1200 mV/RHE, and the results are shown in Figure 2d. Following the previously described treatments (dealloying and annealing), the new CV resembled that of Cu–Pt alloys with Pt‐atoms at the surface [67], with no further de‐alloying peaks detected. The voltammogram demonstrates an oxidative peak at ∼880 mV/RHE related to the adsorption of hydroxyl groups on Pt atoms and the corresponding reductive peak at ∼770 mV/RHE, associated with the desorption of these species and the subsequent ORR. Further, at lower potential values (0.03–0.3 V/RHE), the HUPD‐like features are clearly visible and associated with the concurrent formation of H_2_O_2_ [68, 69].

**FIGURE 3:**
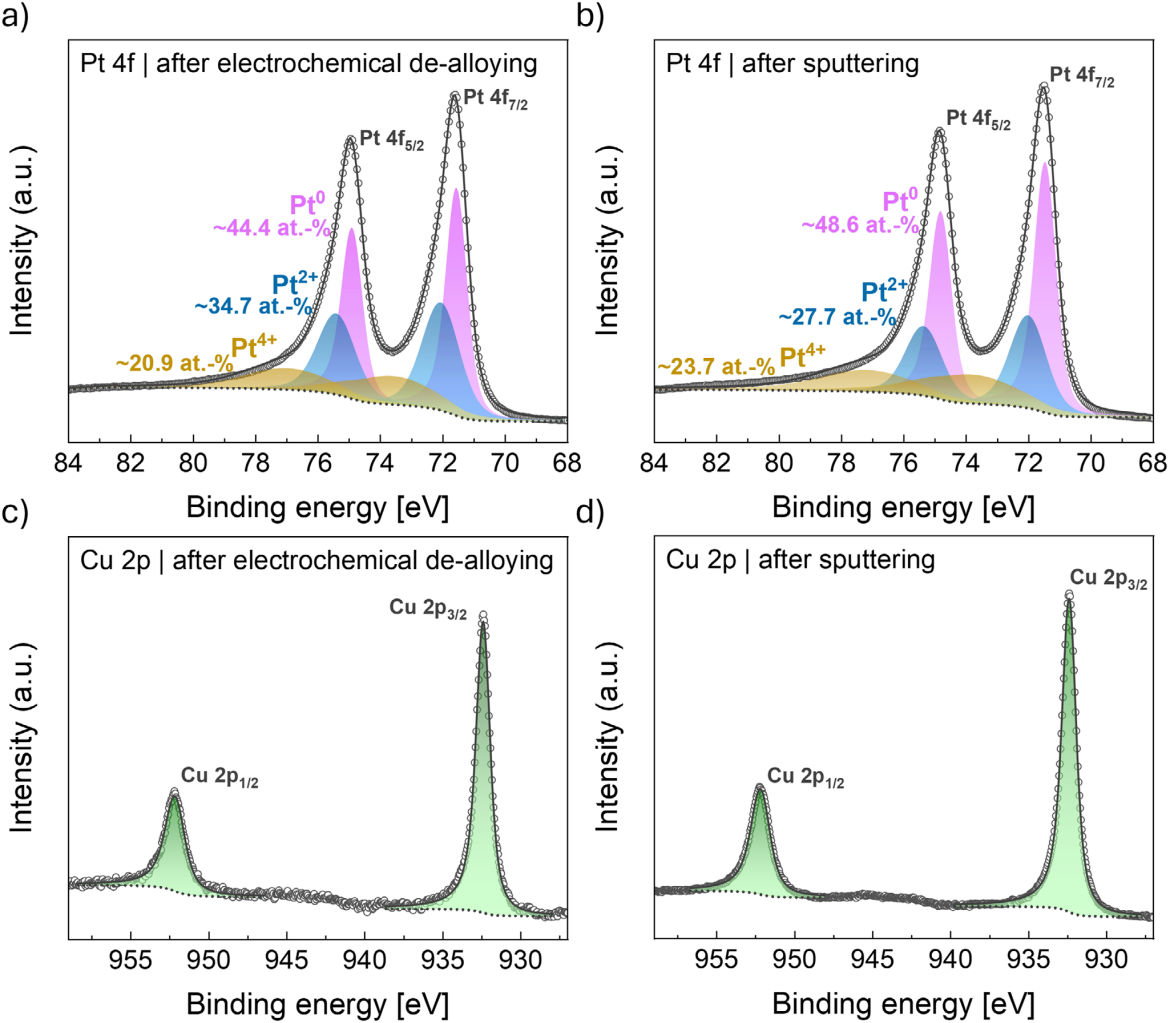
Fitted XPS spectra of the Pt 4f core level regions of the Cu–Pt sample a) after electrochemical cycling and b) after sputtering. Fitted XPS spectra of the Cu 2p core‐level regions of the Cu–Pt sample c) after electrochemical cycling and d) after sputtering. Sputtering with Argon‐ions is used to establish the initial catalyst state before EC‐STM measurements.

Compositional information on the Cu–Pt electrode's surface was obtained via XPS, as depicted in Figure 3. Since XPS only probes the chemical composition of the sample surface and subsurface layers (about 10 nm information depth [70]), it allows for assessing surface transformations when subjected to electrochemical treatments. The Pt 4f core level regions shown in Figure 3a,b reveal only minor changes in the Pt oxidation state ratio between the Ar‐sputtered sample and after the electrochemical treatment. The same holds for the Cu 2p core level regions in Figure 3c,d. Both spectra suggest the presence of metallic Cu, which is in agreement with the Cu LMM spectrum shown in Figure SI3. However, the atomic ratio between Pt and Cu, as determined by the performed fits, differs significantly between the electrochemically de‐alloyed and sputter‐cleaned states. For the (sub)surface of the Cu–Pt sample in the sputter‐cleaned samples, a Pt:Cu ratio of ∼8:1 was estimated, while it increases to ∼13:1 after electrochemical treatment. Therefore, it can be assumed that Cu was removed from the surface according to the commonly observed dealloying process of the less noble Cu metal [45, 46, 47]. Moreover, the presence of Cu in both states suggests that Cu is still incorporated into the sample in subsurface layers after electrochemical measurements, thus confirming the formation of a Pt‐rich overlayer after de‐alloying.

After thoroughly validating the sample's surface morphology and chemical integrity, a first look at the electrochemical activity can be taken. Two main reactions were investigated. Those are the oxygen reduction reactions that result in pure water only and the concurrent formation of H_2_O_2_.

**FIGURE 4:**
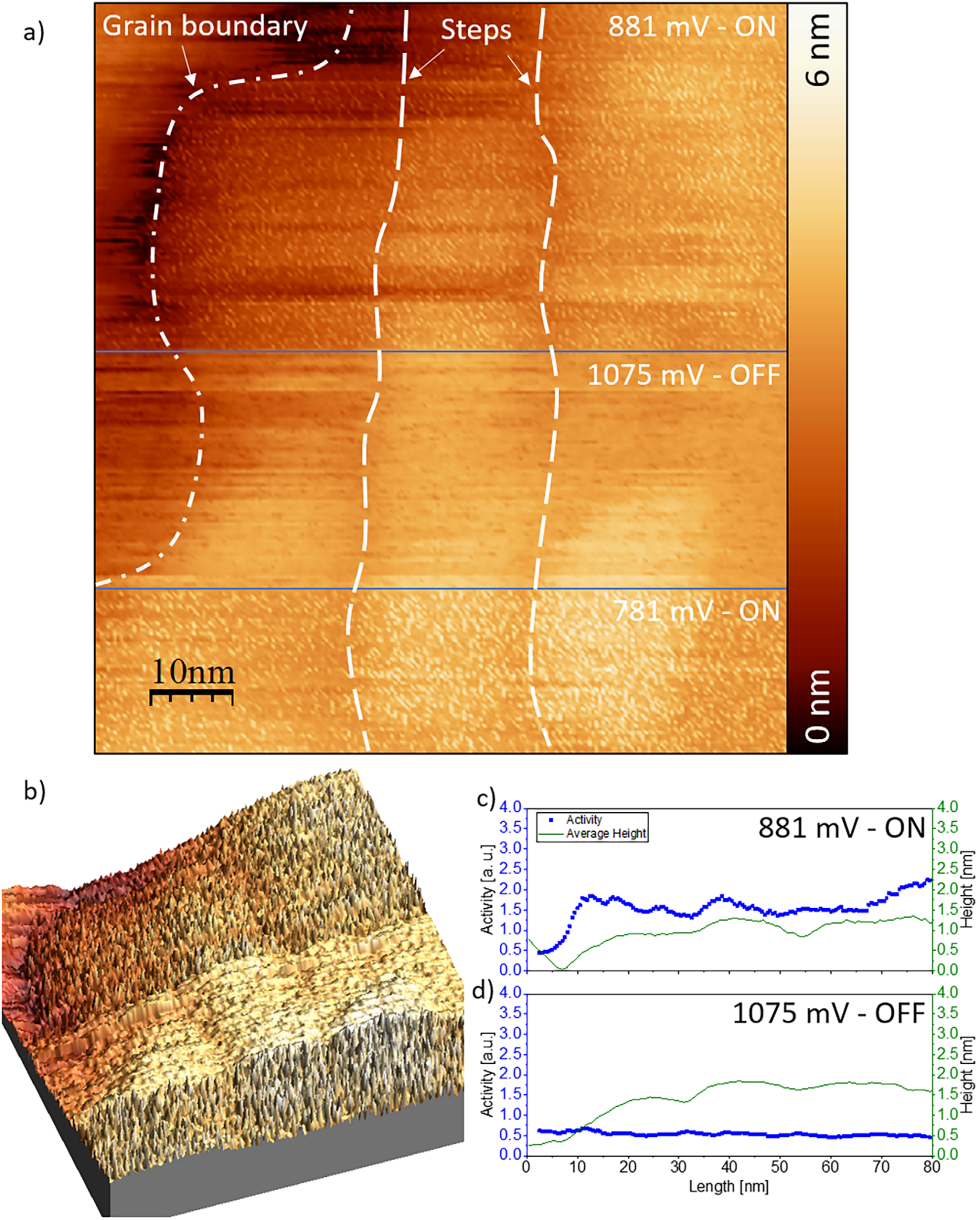
A typical n‐EC‐STM image of the sample under the ORR conditions and when the reaction does not take place. a) Main STM image. The surface is visualized at different potentials vs. RHE, where only water is the reaction product. b) A 3D visualization of the same region. c) & d) Activity plots of the ON and OFF sections shown in a).

An overview of the ORR measurement is presented in Figure 4. The surface is visualized while applying three different potential values. These are an “OFF”‐potential at 1075 mV vs. RHE, where it is considered that no electrochemical reactions are taking place, as well as two lower potentials of 881 and 781 mV vs. RHE, respectively. It can generally be expected that the ORR is already present at these potentials, leading to the formation of H_2_O molecules. The recorded STM image confirms this, as the surface shows small noise features in the “reaction ON” case. These noise features can be seen both in the main STM overview image in Figure 4a and in the 3D representation shown in Figure 4b. It should be noted that no noise can be detected if the reaction is “OFF”, which is to be expected, as no electrochemical reactions should occur under these conditions.

The figures mentioned above reveal several morphological surface features that are of interest. These are: (i) a grain boundary and (ii) small‐scale steps that go vertically through the image. One question that arises is whether these different surface features exhibit increased electrochemical activity or if the main terraces are the most significant contributors to the overall activity. It can already be seen in the raw data in Figure 4a,b that no explicitly intense noise “anomalies” are detectable. Instead, the noise appears to be evenly distributed across most of the surface. The grain boundary, instead, does not show any indication of electrocatalytic activity. Furthermore, a statistical analysis can be conducted to measure the activity locally after each nanometer. This is necessary to quantify the noise and leads to a mathematically based analysis. This tool's summary and working principles were previously reported [61]. Such an analysis was conducted here as well, which is illustrated in Figure 4c,d. These plots show no activity if no surface reaction is taking place. The Full‐Width‐Half‐Maximum (FWHM) values remain relatively constant around their average values. The small‐scale step, as well as other surface features, do not change the activity drastically under the ORR conditions, and hence, after this thorough analysis, it can be concluded that the main terraces on top of the grains act as the most active sites and that defect structures like steps and grain boundaries do not demonstrate increased electrocatalytic activity compared to the terraces.

At much lower electrode potentials, a concurrent electrochemical reaction can be expected. The formation of hydrogen peroxide starts at about 200 mV vs. RHE. The hydrogen evolution reaction eventually starts at a couple of tenths of mV above the 0‐value of the RHE scale, as the electrolyte is saturated with air. Nevertheless, it should be remembered that the ORR does not stop at these lower potentials. It is still present and overlapping with the concurrent formation of water and H_2_O_2_, leading to a situation in which both electrochemical reactions contribute to the measured noise.

**FIGURE 5:**
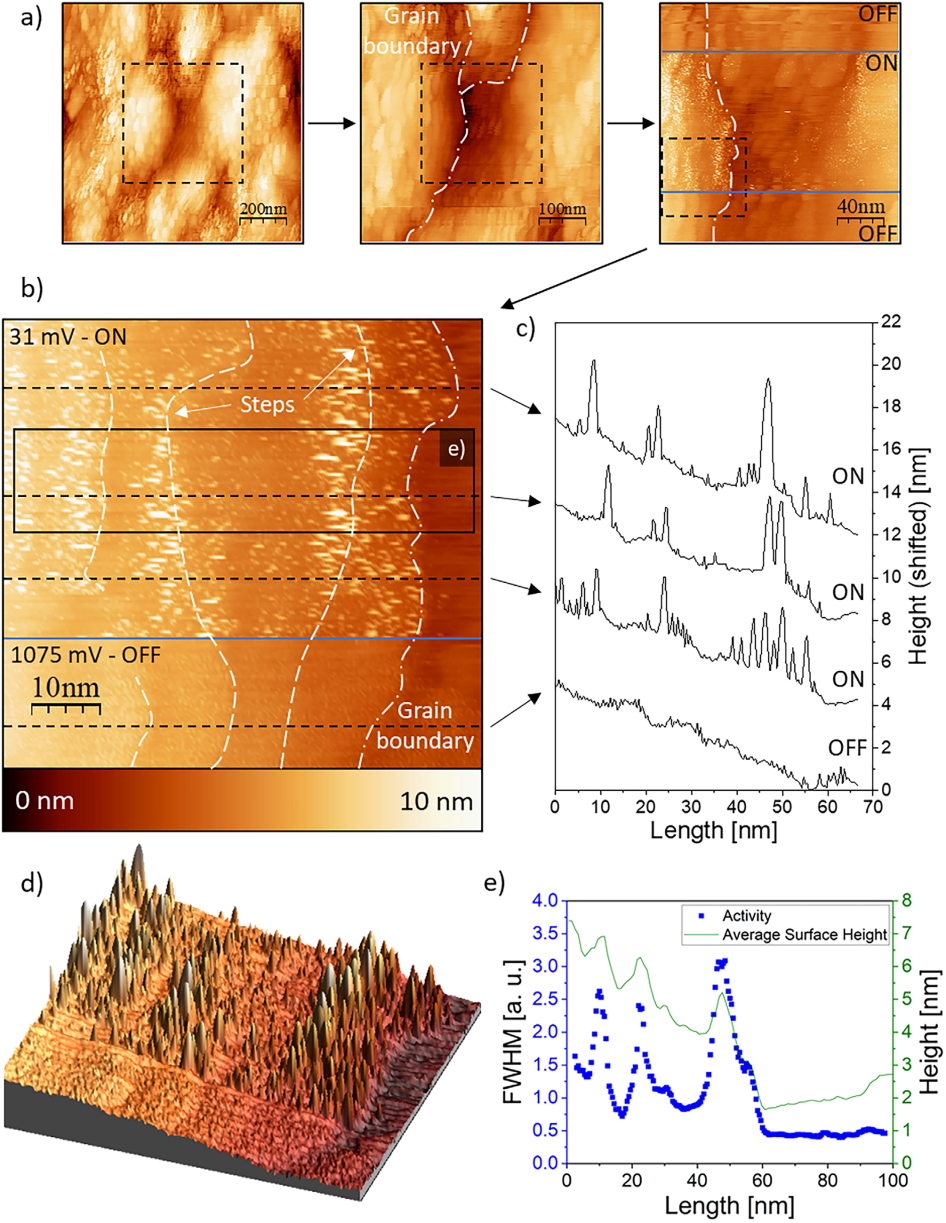
Typical n‐EC‐STM images of the sample under the conditions when H_2_O and H_2_O_2_ are formed simultaneously and when the reaction does not occur. a) An overview at different scales. b) n‐EC‐ STM image taken at the potentials of 31 and 1075 mV vs. RHE. The further analyzed area shown in sub‐Figure e is marked in a black rectangle. c) Waterfall plot of the STM image. Each linescan is shifted by 4 nm to enable better data representation. d) 3D image of a chosen region. e) Further analysis of the activity along the three steps as described in ref. [61].

An area that includes a grain boundary and several small‐scale steps was chosen to analyze the noise at lower applied potentials. The overall measuring area is presented in Figure 5a, which shows several zoom steps at different scales. The measuring area consists of several grains with a pronounced “hole” in the middle. The third image shown in Figure 5a is the measurement where noise features can be observed when the reaction is “ON”. With a further enlargement step, one can observe the area of interest that compares several surface defect structures. This is shown in Figure 5b. An on‐potential of 31 mV vs. RHE is compared to the off‐potential, which is kept at 1075 mV vs. RHE. It is immediately apparent that three lines of noise run halfway through the image from the top to the place of the potential change. After switching the potential back off, no further noise appears on the surface. Several different line scans are presented in Figure 5c. One can notice that the noise features are located on the small‐scale steps. Two of these steps are located on the left and one on the right. While the two steps on the left run through the entire image and can also be seen in the “OFF”‐line scan, the single right step fades towards the bottom of the image and is only poorly visible in the bottom line scan of Figure 5c. While the steps appear to be the primary site of electrochemical activity, some noise peaks, although less frequent, can also be detected on the main terraces. No activity is detected over the grain boundary.

A 3D visualization of this measurement is also given in Figure 5d. The figure shows the features of this image that have already been discussed, with different levels of activity at different surface sites. Once again, an additional analysis is necessary to gain statistical information. The further analyzed area is marked with a black box in Figure 5b, while the actual result is presented in Figure 5e. Also, here the activity is higher on top of the steps, just as expected from the main image. A different feature that is also expected is the difference in the FWHM value between the different terraces. While the left terraces show at least slightly increased FWHM values, the right terrace beyond the grain boundary is entirely inactive. It should be mentioned again that the four‐electron ORR also takes place within this measurement. Because of this, the measured noise might as well partially originate from this reaction instead. This is likely the case for the noise features seen on the terraces, as this phenomenon was already observed previously when analyzing only the higher potential values. An analysis for this measurement when the reaction is “OFF” is given in FigureSI5.

With this, two main features can be concluded from this image. First, it should be noted that, depending on the selectivity, the ORR exhibits site‐related noise. The small‐scale steps are highly active when the concurrent reaction takes place compared to other surface features, such as the grain boundary. This feature has to be potential‐related, as it is not observable for higher potentials.

**FIGURE 6:**
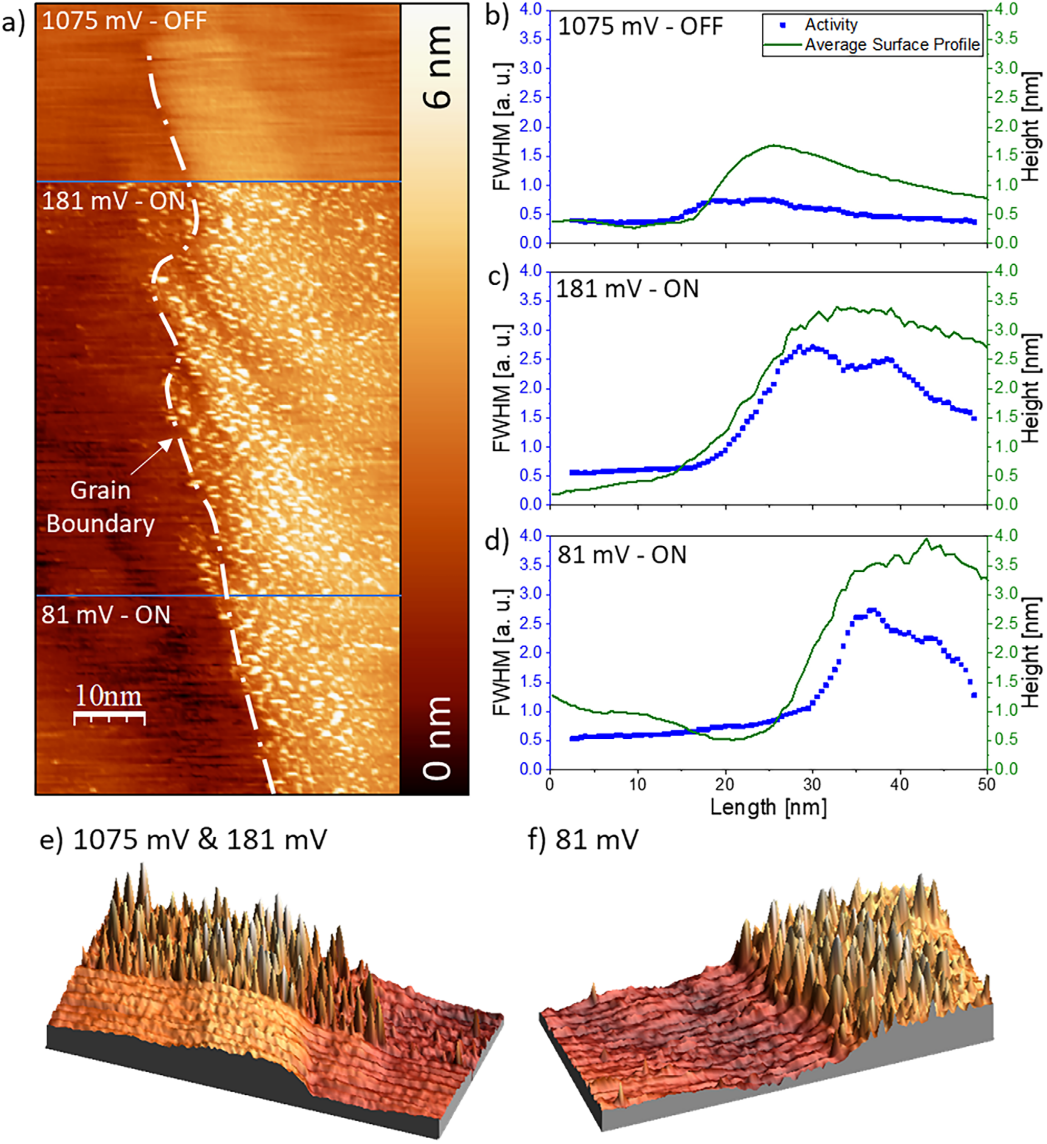
A typical n‐EC‐STM image when the H_2_O_2_ formation is about to start and when the reaction is not active. a) Main STM image. All potential values are given relative to the RHE. b–d) Activity profiles at 1075, 181, and 81 mV vs. RHE, respectively. e) 3D image at potentials of 1075 and 181 mV vs. RHE. f) 3D image at only 81 mV vs. RHE.

Finally, several potential values in the low‐potential regime that belong to the ORR were explored. Potentials of 181 and 81 mV vs. RHE were applied to the surface to study the surface activity at these specific measuring conditions. The main corresponding STM image can be found in Figure 6a. Together with the already analyzed data represented in Figure 5 that was collected at 31 mV, a total of three different potential values are compared. A step defect can be seen through this image and crosses the two featured potentials. The upper terrace just to the right of this step features various noise signals. At first, it seems that the noise might be evenly distributed. However, after a cautious revisit, it is possible to note that the higher noise level is observed close to the step and that the noise decreases further to the right. Figure 6b–d show the activity profile for the measurement at an applied potential of 1075 mV, where no reaction is expected, as well as the activity profiles for 181 and 81 mV, where the ORR resulting in H_2_O_2_ is partially expected. It can be noted that the very first activity profile for the off‐potential is fairly flat and no extraordinary activity spikes can be detected. This changes for an applied surface potential of 181 mV, where an activity peak can be located at the step. Interestingly enough, this is not a clear spike; instead, the activity values remain higher, further away from this step. While they evidently decrease as the distance to the step increases, they do not reach the low‐level values of 1 a. u. or less, as expected from the left terrace. At a lower potential of only 81 mV, this phenomenon still occurs, but it is much less severe. Here, the activity drastically increases at the step, stays high for a couple of nanometers, and then decreases again on the far right side. It should be noted that this phenomenon is not observable at even lower potentials of 31 mV, as shown in Figure 5. At potential levels where the H_2_O_2_ formation is just about to start, both electrochemical reactions might occur at a similar rate. Because of this, both effects, an increase in activity at the steps and an increased activity value on the terraces, are to be expected. Once the potential decreases further, the ORR producing hydrogen peroxide takes over and becomes significantly more dominant. Furthermore, Figure 6e,f show 3D visualizations of the analyzed areas. If observed closely, the described noise behavior can also be detected in these images. The noise in Figure 6e is highly present at the step and on the top terrace, further away from the step.

It was shown that the analyzed CuPt sample exhibits a shift in the specific location of it's active sites if different potentials are applied for the ORR. These potentials can be matched to the selective two and four electron oxygen reduction, producing either hydrogen peroxide or water, respectively. The selectivity of an electrochemical reaction is crucially important and depends on many different reaction parameters, such as the applied surface potential and the nanoscale structure of the active site. It is thought that both parameters influence certain intermediate steps, for example, the oxygen dissociation which is important for the overall intermediate pathway. If hydrogenation steps are energetically more favorable instead, the reaction will take the intermediate pathway via hydrogen peroxide. This might lead to hydrogen peroxide being desorbed prematurely, now serving as the final product. Such a situation seems to be the case for the investigated step defects on the CuPt(111) surface for potentials lower than about 200 mV [71, 72, 73].

The topic of the precise intermediate reaction pathway may also not be entirely solved by theoretical literature considerations, as these are not consistent with each other. Some groups claim a dissociative pathway [72] while others promote the idea of oxygen dissociation via hydrogen peroxide intermediates [74, 75]. However, all studies agree that CuPt is generally a better ORR catalyst than pure platinum.

## 4 Conclusions

A Cu–Pt alloy thin film surface was investigated concerning its electrochemical activity and selectivity for the oxygen reduction reaction. The findings indicate that the ORR primarily occurs on the terraces in the case of the four‐electron pathway. Notably, the grain boundaries themselves do not contribute to the ORR activity.

When the electrode potentials are shifted to the region where H_2_O_2_ formation is also possible, the type of the most active sites changes. These manifest themselves at atomic‐scale defects, namely steps. While terraces also show a small but non‐zero activity during the measurements, this is likely due to concurrent four‐electron reaction processes at the applied potentials. Once again, terrace activity differences can be detected, while grain boundaries do not contribute to the activity. These findings can be used in designing the shape of nanoparticles for improved nanostructured materials, e.g., in energy applications.

## Conflicts of Interest

The authors declare no conflicts of interest.

## Supporting information




**Supporting File**: smtd70496‐sup‐0001‐SuppMat.docx.

## Data Availability

The data that support the findings of this study are available from the corresponding author upon reasonable request.
