# Supplementary material for: Local Activity and Selectivity Hotspots in Cu‐Pt Model Thin‐Film Electrocatalysts for Oxygen Reduction
Source: Small Methods. 2026 Jan 28;10(4):e02169. doi: 10.1002/smtd.202502169 (PMC12929926; doi:10.1002/smtd.202502169)
Supplement: Supplementary file 1 — Supporting File: smtd70496‐sup‐0001‐SuppMat.docx. [file SMTD-10-e02169-s001.docx]

**Supporting Information**

**Local Activity and Selectivity Hotspots in Cu-Pt Model Thin-Film Electrocatalysts for Oxygen Reduction**

*Lewin V. Deville,^1^ Rico Zehl,^2^ Luca Saluta,^1^ Qingdian Liao,^1^ Peter M. Schneider,^1^ Tobias Piotrowiak,^2^ Benedikt Kohnen,^2^ Ellen Suhr,^2^ Alfred Ludwig,^2,^* Aliaksandr S. Bandarenka^1,3,^**

1 - Physics of Energy Conversion and Storage, Technical University of Munich, Physics Department, James-Franck-Str. 1, 85748 Garching, Germany

2 - Ruhr University Bochum, Institute for Materials, Chair for Materials Discovery and Interfaces, Universitätsstr. 150, 44780 Bochum, Germany

3 - Catalysis Research Center TUM, Ernst-Otto-Fischer-Str. 1, 85748, Garching, Germany

*E-mail: [alfred.ludwig@rub.de](mailto:alfred.ludwig@rub.de), [bandarenka@ph.tum.de](mailto:bandarenka@ph.tum.de)

| 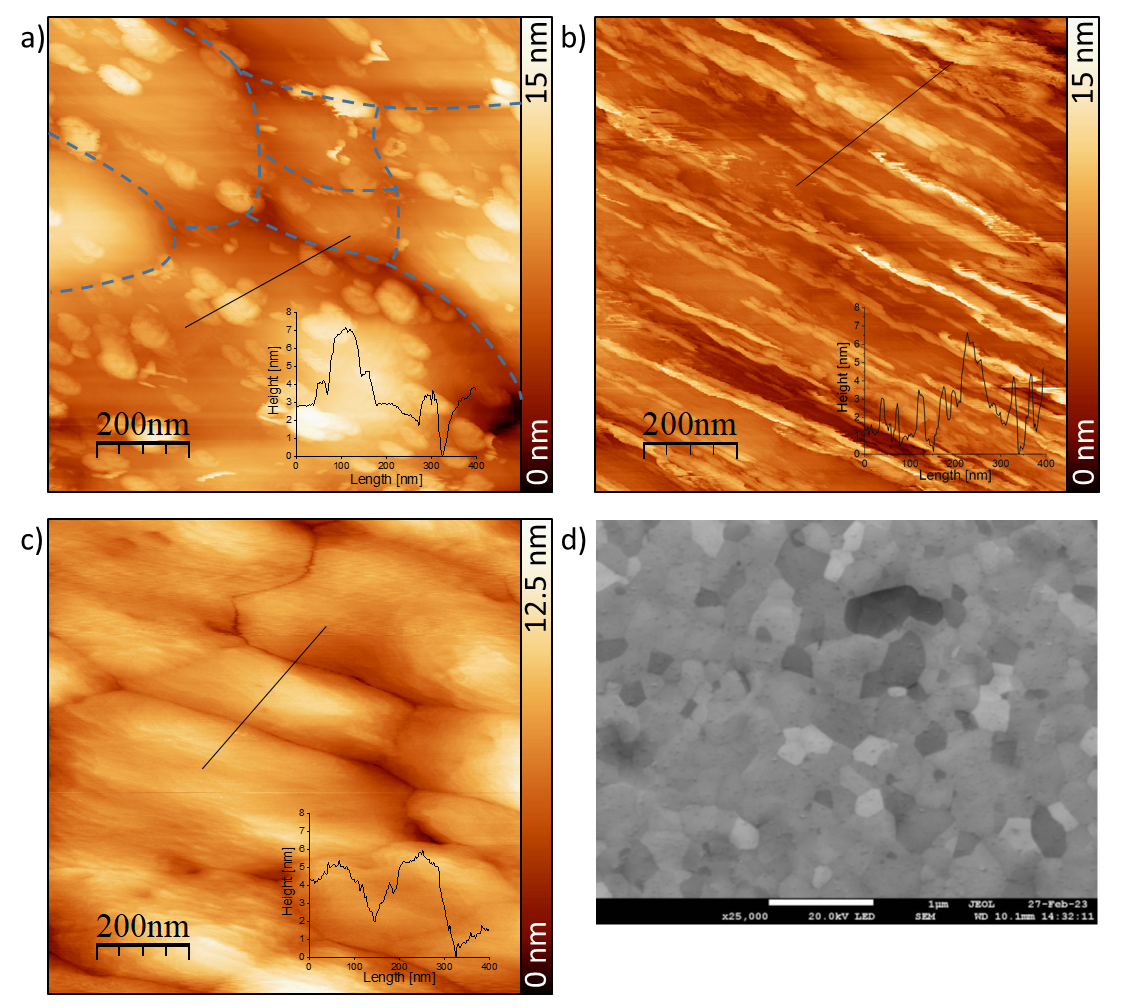 |
| --- |
| ***Figure SI1****: Overview of the dealloying and annealing process. a) First sample overview prior to any engagements. Note the slight boundary structures, but also the impurities on the surface. b) Surface structure after dealloying. Note a lack of any grain boundaries. c) Final surface structure after dealloying and annealing of the surface. The expected grain structure is visible again. Furthermore, most impurities seem to have disappeared. d) A typical SEM image showing the grain structure.* |

| 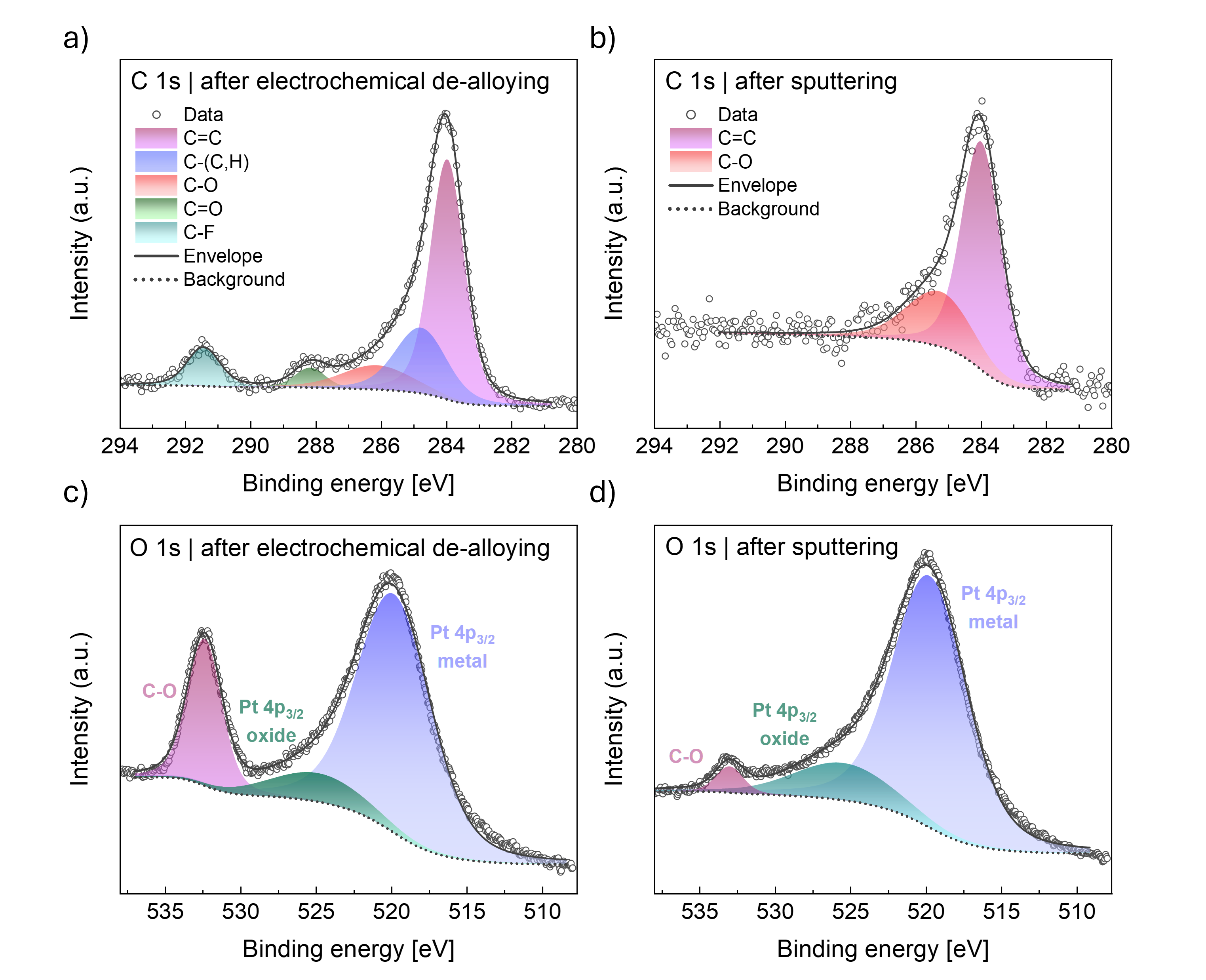 |
| --- |
| ***Figure SI2****: Fitted XPS spectra of the C 1s core level regions of the Cu-Pt sample a) after electrochemical de-alloying and b) after sputtering. Fitted XPS spectra of the O 1s core-level regions of the Cu-Pt sample c) after electrochemical de-alloying and d) after sputtering. Sputtering with Argon ions is used to establish the initial catalyst state before EC-STM measurements.* |

| 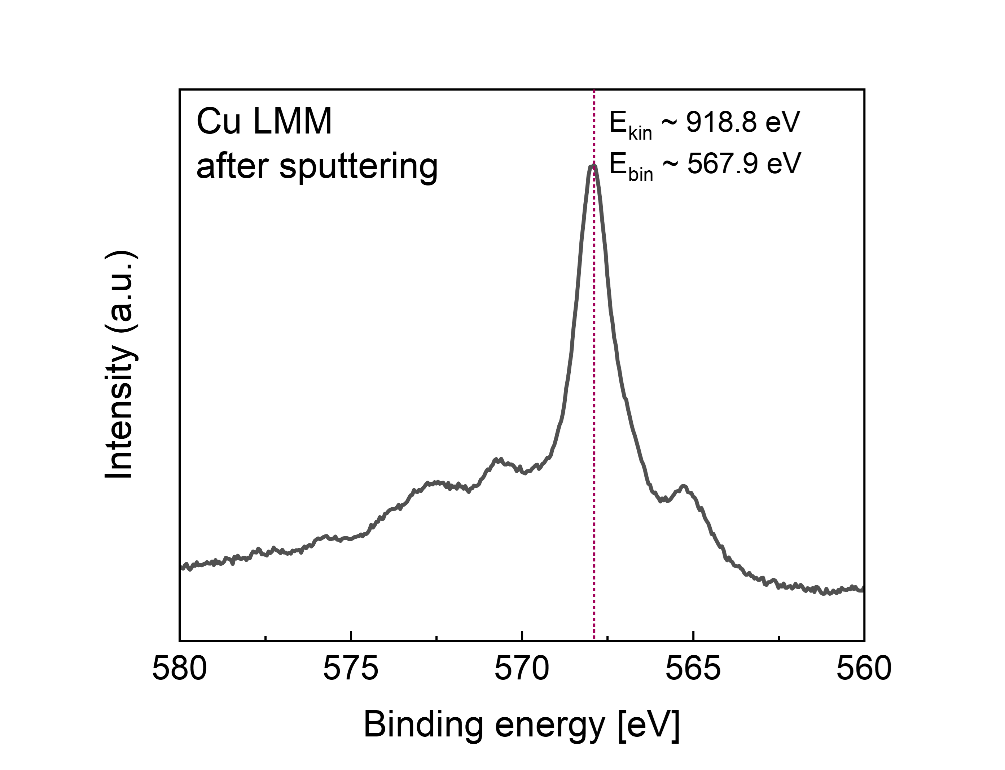 |
| --- |
| ***Figure SI3****: Cu LMM region of the Cu-Pt sample a) after sputtering. The vertical dotted line marks the peak position (given in binding and kinetic energy), which is in agreement with metallic Cu. Sputtering with Argon ions is used to establish the initial catalyst state before EC-STM measurements.* |

| 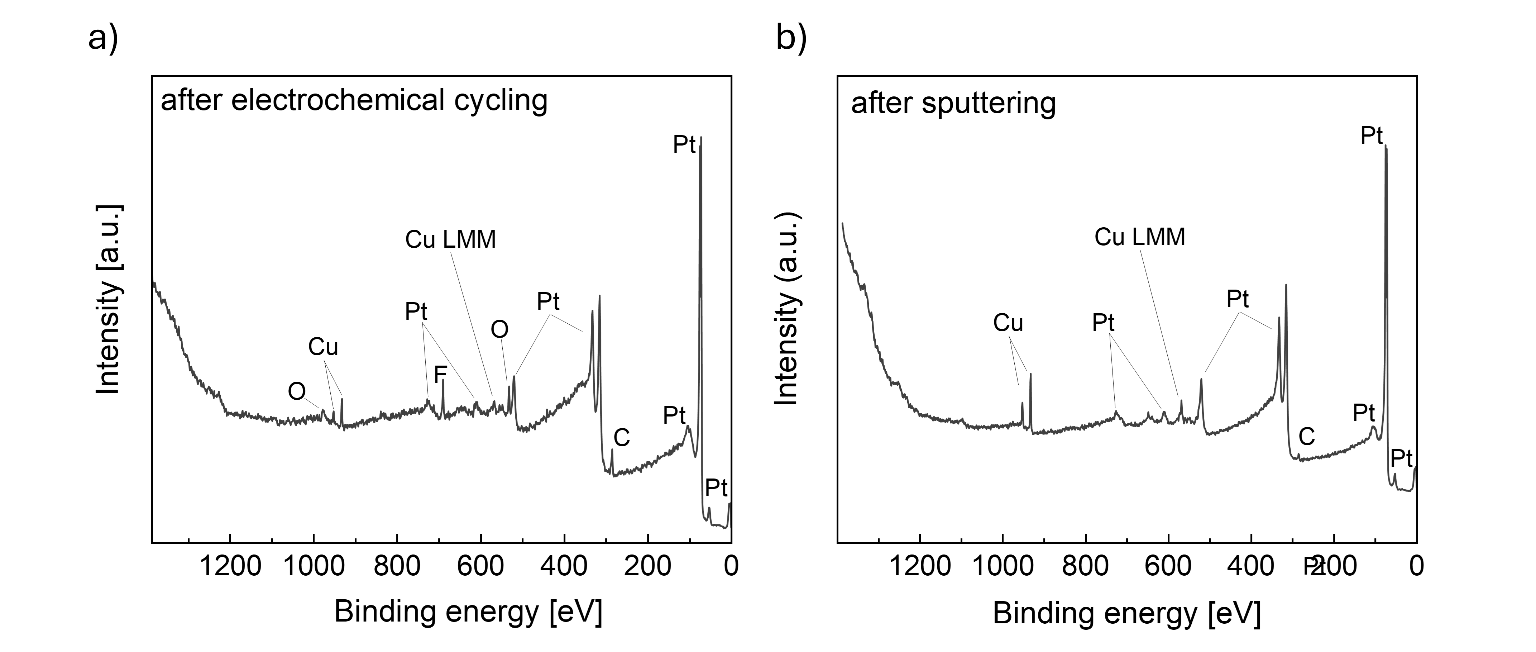 |
| --- |
| ***Figure SI4****: Survey spectra of the Cu-Pt sample a) after electrochemical de-alloying and b) after sputtering. Elements present at the (sub)surface layers are indicated. Sputtering with Argon ions is used to establish the initial catalyst state before EC-STM measurements.* |
| 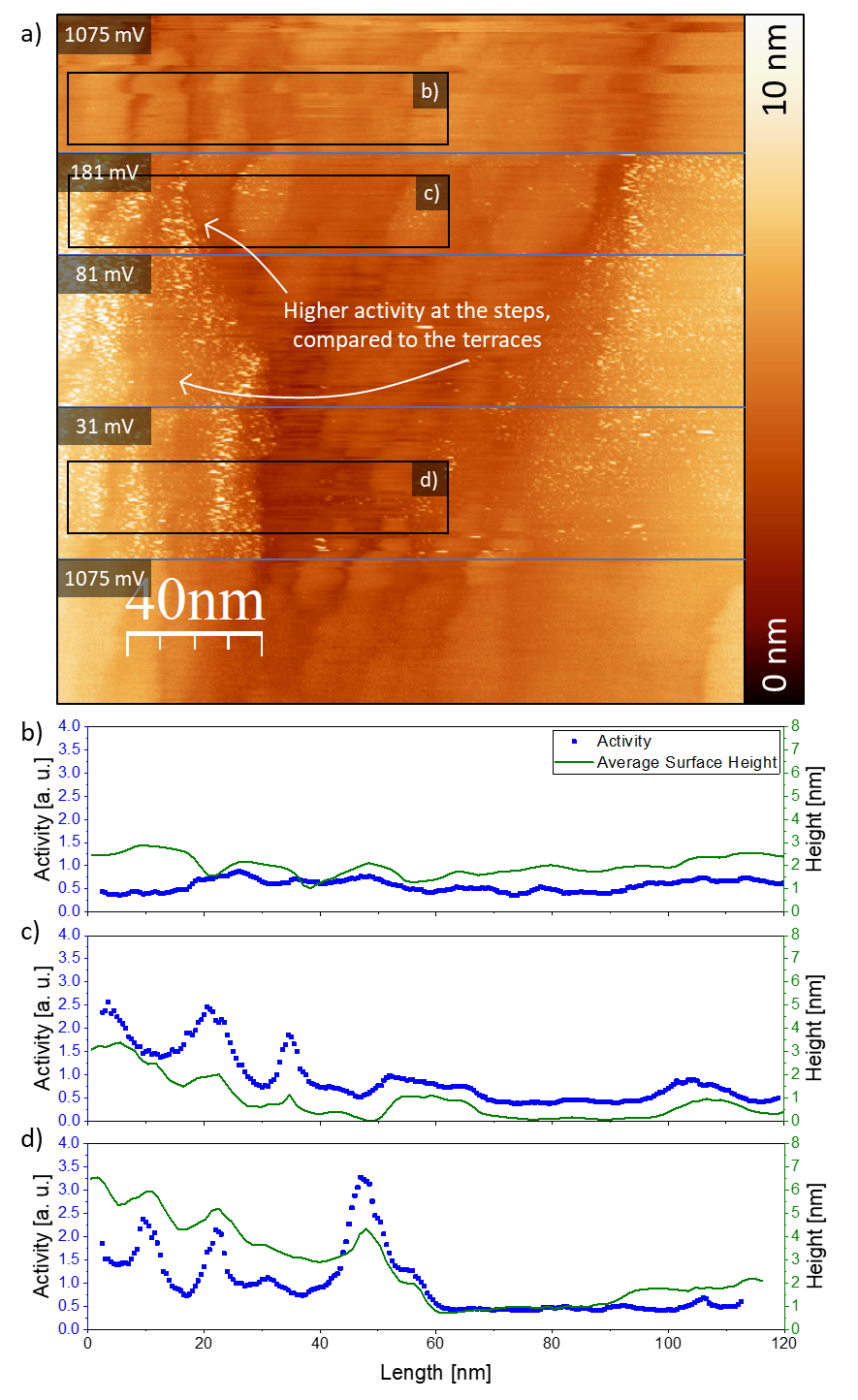 |
| ***Figure SI5****: A further look at the Proton reduction reaction at lower potentials. a) Overview image of the scanned surface. All potential values are relative to RHE. Three different “on” potentials and one “off” potential are measured. b) Further analysis of the “off” potential. The activity curve stays fairly flat, as expected. c) A measurement at 181 mV vs RHE. Surface steps have a high contribution to the measured noise. Furthermore, some selected terraces seem to be active as well. d) Final analysis at 31 mV vs RHE, also shown in the main manuscript. Main activity at the surface steps. The terraces are only slightly active.* |
